# A Systematic Review and Meta-Analysis of the Effect of Pentagalloyl Glucose Administration on Aortic Expansion in Animal Models

**DOI:** 10.3390/biomedicines9101442

**Published:** 2021-10-11

**Authors:** Jonathan Golledge, Shivshankar Thanigaimani, James Phie

**Affiliations:** 1 Queensland Research Centre for Peripheral Vascular Disease, College of Medicine and Dentistry, James Cook University, Townsville, QLD 4810, Australia; shiv.thanigaimani@jcu.edu.au (S.T.); james.phie@jcu.edu.au (J.P.); 2 The Department of Vascular and Endovascular Surgery, The Townsville Hospital, Townsville, QLD 4810, Australia; 3 The Australian Institute of Tropical Health and Medicine, James Cook University, Townsville, QLD 4810, Australia

**Keywords:** pentagalloyl glucose, abdominal aortic aneurysm, aortic aneurysm

## Abstract

Background: The aim of this systematic review was to pool evidence from studies testing if pentagalloyl glucose (PGG) limited aortic expansion in animal models of abdominal aortic aneurysm (AAA). Methods: The review was conducted according to the PRISMA guidelines and registered with PROSPERO. The primary outcome was aortic expansion assessed by direct measurement. Secondary outcomes included aortic expansion measured by ultrasound and aortic diameter at study completion. Sub analyses examined the effect of PGG delivery in specific forms (nanoparticles, periadventitial or intraluminal), and at different times (from the start of AAA induction or when AAA was established), and tested in different animals (pigs, rats and mice) and AAA models (calcium chloride, periadventitial, intraluminal elastase or angiotensin II). Meta-analyses were performed using Mantel-Haenszel’s methods with random effect models and reported as mean difference (MD) and 95% confidence intervals (CIs). Risk of bias was assessed with a customized tool. Results: Eleven studies reported in eight publications involving 214 animals were included. PGG significantly reduced aortic expansion measured by direct observation (MD: −66.35%; 95% CI: −108.44, −24.27; *p* = 0.002) but not ultrasound (MD: −32.91%; 95% CI: −75.16, 9.33; *p* = 0.127). PGG delivered intravenously within nanoparticles significantly reduced aortic expansion, measured by both direct observation (MD: −116.41%; 95% CI: −132.20, −100.62; *p* < 0.001) and ultrasound (MD: −98.40%; 95% CI: −113.99, −82.81; *p* < 0.001). In studies measuring aortic expansion by direct observation, PGG administered topically to the adventitia of the aorta (MD: −28.41%; 95% CI −46.57, −10.25; *p* = 0.002), studied in rats (MD: −56.61%; 95% CI: −101.76, −11.46; *p* = 0.014), within the calcium chloride model (MD: −56.61%; 95% CI: −101.76, −11.46; *p* = 0.014) and tested in established AAAs (MD: −90.36; 95% CI: −135.82, −44.89; *p* < 0.001), significantly reduced aortic expansion. The findings of other analyses were not significant. The risk of bias of all studies was high. Conclusion: There is inconsistent low-quality evidence that PGG inhibits aortic expansion in animal models.

## 1. Introduction

Abdominal aortic aneurysm (AAA) rupture is estimated to be responsible for approximately 200,000 deaths per year worldwide [1]. The only current treatments for AAA are open or endovascular surgical repair [2,3]. Randomized controlled trials have suggested that the surgical repair of small AAAs (<55 mm) does not reduce mortality [4]. Clinical guidelines recommend that small asymptomatic AAAs are treated conservatively [2,3]; however, up to 70% of non-surgically treated AAAs continue to grow in size, thereby increasing the risk of rupture [5]. A drug therapy for small AAAs would be of great clinical value.

Past preclinical and clinical AAA research has focused on testing drugs that reduce aortic inflammation, inhibit extracellular matrix degradation or lower blood pressure [6,7,8]. Despite hundreds of preclinical studies and multiple clinical trials, none of these drugs have come into routine clinical practice for treating AAA [6,7]. Pentagalloyl glucose (PGG) is a polyphenolic derivate of tannic acid that is currently under investigation as a treatment to stabilize AAA [9]. PGG has been proposed to reduce the turnover of collagen and elastin by cross-linking these key extracellular matrix proteins [9]. A growing number of studies have examined the effect of PGG administration on aortic expansion in animal models of AAA. Many of these studies have reported reduced aortic expansion [10,11,12,13]. However, a recent study reported no effect in two rodent models [14].

Given the conflicting findings of these animal studies and since PGG is now being tested as a treatment for small AAA in patients, a critical review of the past preclinical evidence is needed. The aim of this study was to undertake a systematic review and meta-analysis by pooling data from studies testing the effect of PGG on aortic expansion in animal models of AAA.

## 2. Methods

### 2.1. Search Strategy and Eligibility Criteria

This systematic review was conducted according to the Preferred Reporting Items for Systematic Reviews and Meta-Analyses (PRISMA) statement and was registered in the PROSPERO database (Registration number: CRD42021275777) [15]. The PubMed and Web of Science (via ISI Web of Knowledge; 1965) databases were searched from inception to 14 September 2021. The search string ((“Pentagalloyl”[All Fields] AND (“glucose”[MeSH Terms] OR “glucose”[All Fields] OR “glucoses”[All Fields] OR “glucose s”[All Fields])) OR “PGG”[All Fields]) AND (“AAA”[All Fields] OR (“aneurysm”[MeSH Terms] OR “aneurysm”[All Fields] OR “aneurysms”[All Fields] OR “aneurysm s”[All Fields] OR “aneurysmal”[All Fields] OR “aneurysmally”[All Fields] OR “aneurysmic”[All Fields])) was used. No language or date restrictions were used. Reference lists of the studies identified were also searched. Eligibility criteria for inclusion were: an animal study involving any AAA model testing the effect of PGG on aortic diameter increase; aortic diameter reported at a minimum of one time point after PGG administration; and inclusion of a control group not receiving PGG but otherwise receiving similar care. Studies including animals receiving PGG but not reporting aortic diameter, or where this could not be extracted or obtained from the authors, were excluded. In vitro or ex vivo studies were also excluded.

### 2.2. Data Extraction

The primary outcome was relative increase in the maximum diameter of the aorta after PGG administration, as compared to controls not receiving PGG, reported as percentage. This was required to be measured by direct observation by analysis of the in situ aortas at laparotomy, or the excised aortas using calipers or pictures. Secondary outcomes were aortic expansion measured by ultrasound, final maximum AAA diameter reported in millimeters, and AAA incidence and aortic rupture reported as numbers and percentage in mice allocated to PGG compared to controls. Other data extracted included: the types of AAA models; animal age, sex and strain; sample sizes; method of aortic diameter measurement; definition of AAA incidence; days after AAA induction that PGG or control were first administered; duration over which aortic expansion was studied; PGG form, dose and route of administration; and the findings of histological, biochemical and biomechanical studies. Data were extracted by three authors separately and inconsistencies were resolved through discussion. In studies where aortic diameters were reported only in graphs, they were extracted using ImageJ 64-bit version 1.8.0_172 (National Institute of Health, Bethesda, MD, USA).

### 2.3. Risk of Bias

A risk of bias tool was developed by combining the Systematic Review Centre for Laboratory Animal Experimentation (SYRCLE) and a previously developed risk of bias tool for AAA model research [16,17]. This incorporated the first nine questions of the SYRCLE tool and four questions from the AAA model risk of bias tool. These additional questions were focused on: the justification of the dose of PGG used; sample size estimation; whether aortic diameter was reported at first allocation to PGG or control and at study completion; and the reproducibility of aortic diameter measurement. Risk of bias was assessed by three authors and differences were resolved by discussion. The scores of the finally agreed upon risk of bias assessment were summed and reported as a percentage. The studies were rated as high (<50%), medium (51–70%) or low (71–100%) risk of bias.

### 2.4. Data Analysis

Meta-analyses were planned to be performed for any of the primary and secondary outcomes if data were reported in at least two studies. Sub analyses were also planned, and limited to studies using similar modes of PGG administration (nanoparticle incorporated, aortic periadventitial, or intraluminal); separating treatment starting at the time AAA induction commenced (i.e., testing effect on AAA development) versus starting after AAA had been established for at least one day (i.e., testing effect on AAA growth); performed in the same animals species (e.g., pigs, mice and rats), or AAA model types (calcium chloride, periadventitial, intraluminal elastase or angiotensin II); and excluding studies deemed to be at high risk of bias [18]. A leave-one-out-sensitivity analysis was performed to assess the contribution of each study to the pooled estimates of the primary outcome by excluding individual studies one at a time and recalculating the pooled estimates [19]. All meta-analyses were performed using Mantel-Haenszel’s statistical methods and random effect models anticipating substantial heterogeneity [20]. The results were reported as mean differences (MDs), with 95% confidence intervals (CIs), for aortic diameter increase and relative risk (RR) and 95% CIs for AAA incidence and rupture. All statistical tests were two-sided and *p*-values < 0.05 were considered significant. Statistical heterogeneity was assessed using the I^2^ statistic and interpreted as low (0 to 49%), moderate (50 to 74%) or high (75 to 100%) [21]. Publication bias was assessed by funnel plots comparing the summary estimate of each study and its precision (1/standard error) [19]. A minimum of ten studies were required to develop funnel plots to analyze publication bias [19]. Meta-analyses were conducted using ‘meta’ package, and the sensitivity analysis was performed using the ‘dmetar’ package of R program version 4.0.3.

## 3. Results

### 3.1. Included Studies

**Figure 1:**
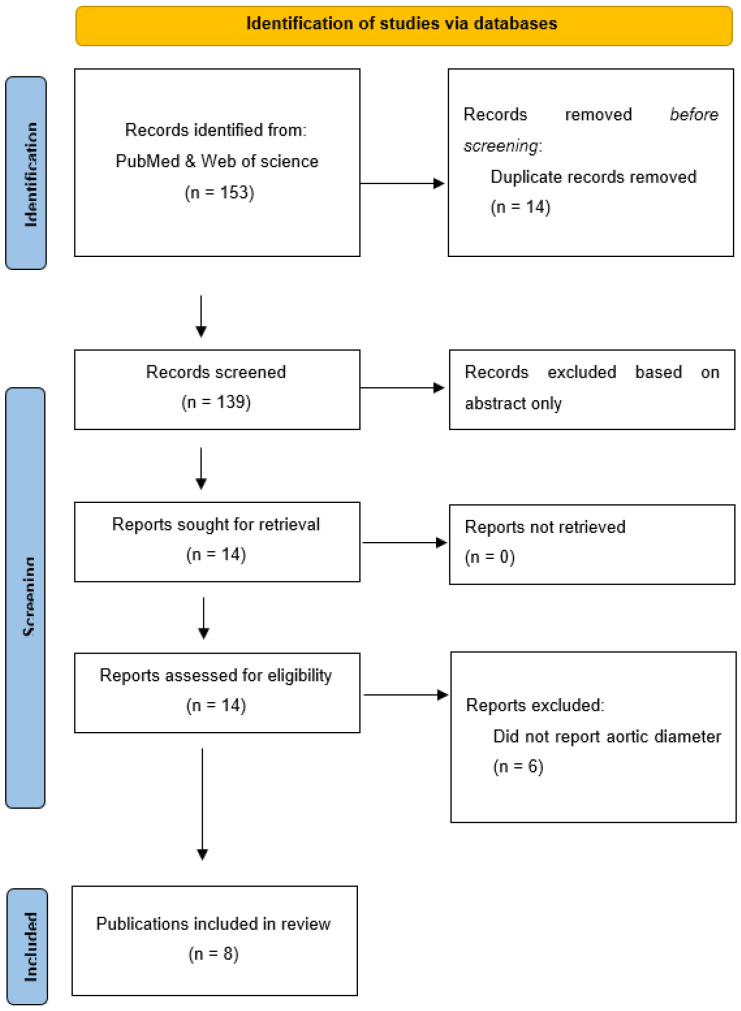
Preferred Reporting Items for Systematic Reviews and Meta-Analyses flow diagram. A total of 153 publications were screened and, after exclusion of irrelevant studies, 8 publications were included.

From 139 unique publications identified by the search, eight publications met the inclusion criteria and provided a total of 11 unique studies (Figure 1). Three publications included two different eligible studies [10,13,22], while the other five publications included one eligible study each [11,12,23,24,25]. Six studies used rats, four used mice and one used pigs (see Table 1). Overall, a total of 214 animals were included, with total sample sizes in individual studies varying from 12 to 30 (Table 1). The AAA models used included periadventitial infrarenal aortic calcium chloride application in five studies, intraluminal infrarenal aortic elastase in three studies (including the addition of aortic balloon dilatation and juxta-renal stenosing cuffs in the pig study) [25], periadventitial infrarenal aortic elastase application in two studies and subcutaneous angiotensin II infusion in one study (Table 1). In six studies, PGG and the control interventions were initiated at the time when AAA induction was commenced, whereas in the other five studies, PGG and the control interventions commenced between 10 and 42 days after AAA induction (see Table 2). Animals were monitored for between 14 and 42 days after the PGG and control interventions commenced (Table 2). The routes, forms and doses of the PGG administered varied (see Table 2). Four studies tested the intravenous delivery of PGG incorporated in nanoparticles, another four studies tested PGG applied topically to the adventitia of the aorta and three studies tested PGG infused into the lumen of the aorta (in one case, this was delivered by a drug-eluting balloon). Nine studies included a vehicle control and no intervention was given to the controls in two studies (see Table 2). All eleven studies reported percentage increases in aortic diameter for both the interventional and the control groups. Measurements were performed by direct observation alone in five studies, ultrasound alone in four studies and both measurement methods in two studies (Table 2). Six studies reported the actual aortic diameter at the end of the study. Measurements were performed by direct observation alone in two studies, ultrasound alone in three studies and both measurement methods in one study (Table 2). Only two studies reported AAA incidence [13,24]. Aortic rupture is not a feature of the models used in most studies, with only one study reporting this outcome [10].

### 3.2. Risk of Bias of Included Studies

All 11 studies were considered to have a high risk of bias with overall scores on the 13 item quality assessment tool ranging between 8% and 31% (see Table 3). Common risks of bias identified were failure to randomize animals to the intervention and control group, failure to blind investigators and outcome assessors, failure to justify PGG dose, absence of sample size rationales and not reporting the reproducibility of aortic diameter measurement (Table 3).

### 3.3. Effect of PGG on Aortic Expansion

**Figure 2:**
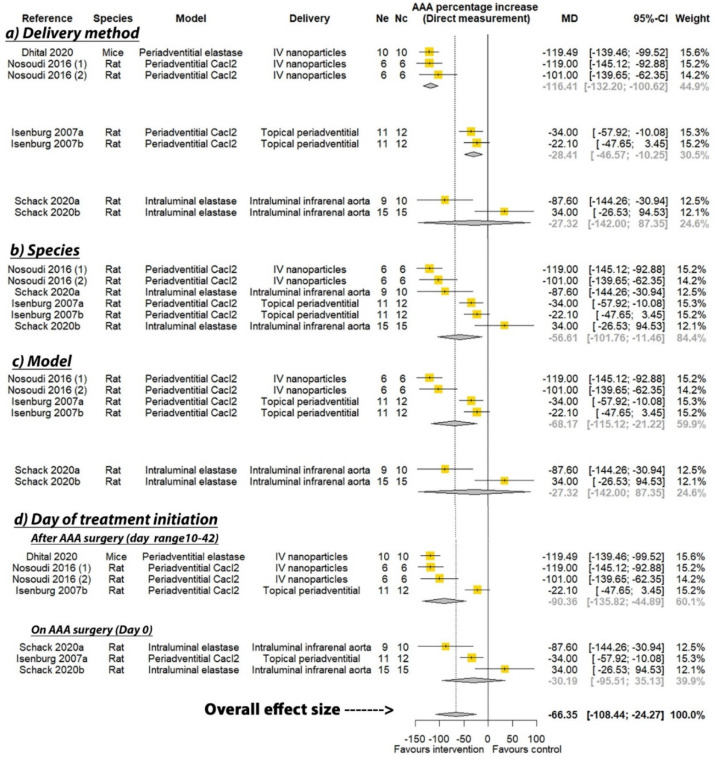
Meta-analysis of studies testing the effect of pentagalloyl glucose on aortic expansion measured by direct observation. MD = Mean difference; Ne = Number of animals in experimental group; Nc = Number of animals in control group; CI = Confidence interval. a/b: Three of the publications included two separate studies that were considered independently.

**Figure 3:**
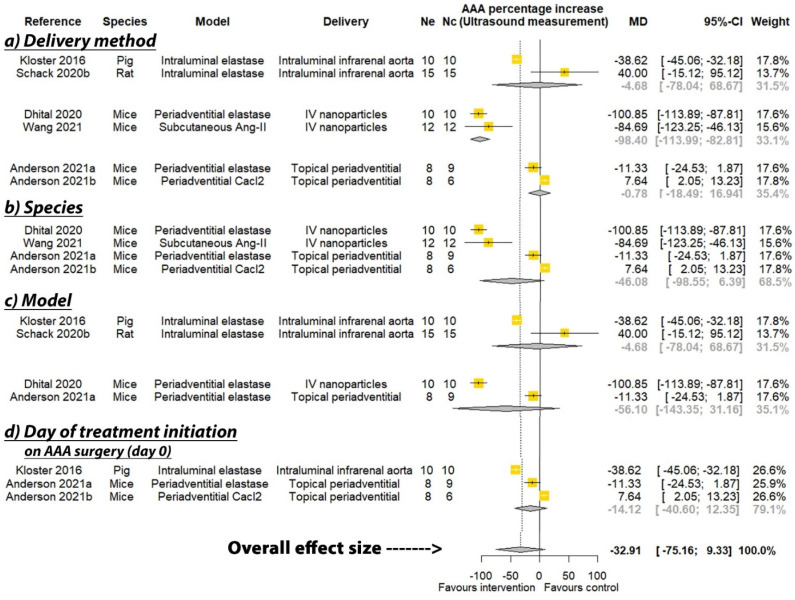
Meta-analysis of studies testing the effect of pentagalloyl glucose on aortic expansion measured by ultrasound. MD = Mean difference; Ne = Number of animals in experimental group; Nc = Number of animals in control group; CI = Confidence interval. a/b: Three of the publications included two separate studies that were considered independently.

PGG was reported to significantly reduce the percentage increase in aortic diameter in six of the seven studies where this was measured by direct observation, and three of the six studies that measured aortic diameter percentage increase by ultrasound (see Table 2). A meta-analysis suggested that PGG significantly reduced aortic expansion when measured by direct observation (MD: −66.35%; 95% CI: −108.44, −24.27; *p* = 0.002), but not ultrasound (MD: −32.91%; 95% CI: −75.16, 9.33; *p* = 0.127), compared to the controls (Figure 2 and Figure 3). In studies measuring aortic expansion by direct observation, PGG administered intravenously through nanoparticles (MD: −116.41%; 95% CI: −132.20, −100.62; *p* < 0.001), topically to the adventitia of the aorta (MD: −28.41%; 95% CI: −46.57, −10.25; *p* = 0.002), studied in rats (MD: −56.61%; 95% CI: −101.76, −11.46; *p* = 0.014), in the calcium chloride model (MD: −68.17%; 95% CI: −115.12, −21.22; *p* = 0.004), and where PGG treatment was initiated after model development on days ranging between 10 and 42 (MD: −90.36; 95% CI: −135.82, −44.89; *p* < 0.001), significantly reduced aortic expansion (Figure 2). A sensitivity analysis of the studies reporting aortic expansion by direct measurement found that the individual removal of any single study did not change the significance of the findings (Supplementary Table S1). In studies measuring aortic expansion by ultrasound measurement, PGG administered intravenously using nanoparticles significantly reduced aortic expansion (MD: −98.40%; 95% CI: −113.99, −82.81; *p* < 0.001) (Figure 3). The findings of other sub analyses were not significant (Figure 3). Funnel plots were not performed, due to data not being available from a minimum number of 10 studies.

### 3.4. Effect of PGG on Final AAA Diameter

**Figure 4:**
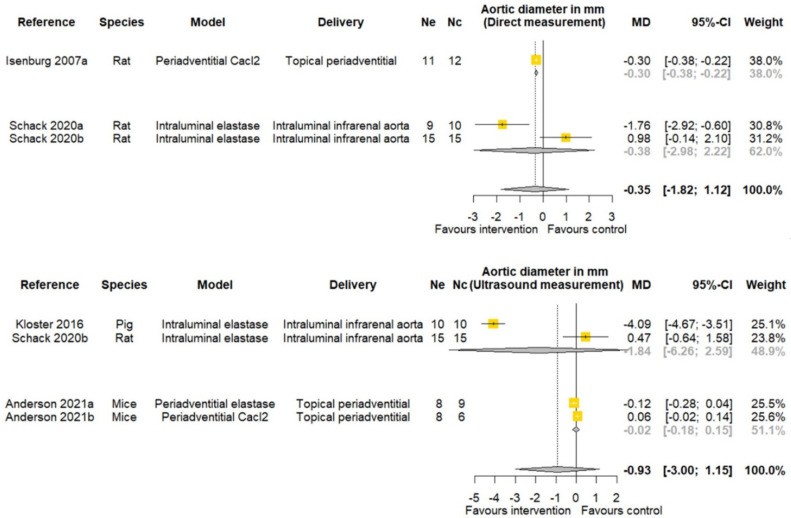
Meta-analysis of studies testing the effect of pentagalloyl glucose on final aortic diameter in animal models of abdominal aortic aneurysm. MD = Mean difference; Ne = Number of animals in experimental group; Nc = Number of animals in control group; CI = Confidence interval. a/b: Three of the publications included two separate studies that were considered independently.

One of three studies reported that PGG significantly reduced AAA diameter measured by direct observation at study completion (Table 2). One of four studies reported that PGG significantly reduced AAA diameter measured by ultrasound at study completion (Table 2). Meta-analyses suggested that PGG did not significantly reduce aortic diameter assessed by both direct measurement (MD: −0.35 mm; 95% CI −1.82, 1.12; *p* = 0.642) and ultrasound (MD −0.93 mm; 95% CI −3.00, 1.15; *p* = 0.381) (Figure 4). The findings of other sub analyses were not significant (Figure 4).

### 3.5. Effect of PGG on AAA Incidence

Two studies reported the incidence of AAA (see Table 2), but only one study initiated PGG treatment on the day of AAA induction, with 66.7% of rats in the control group developing AAA, compared to 18.2% of the rats receiving periadventitial aortic PGG at study completion [13]. Another study found that 100% of rats receiving PGG-loaded nanoparticles delivered intravenously 42 days after AAA induction developed AAA similar to the control group [24]. A meta-analysis of the two studies suggested that AAA incidence was not significantly different between rats receiving PGG and the controls, with large CIs (RR: 0.62; 95% CI: 0.00, 1751.32; *p* = 0.588, Supplementary Figure S1).

### 3.6. Findings from Histological and Molecular Biology Analyses

Histology findings from some studies found that animals receiving PGG had less aortic media elastic fiber degradation, more desmosine content and decreased macrophage infiltration (See Table 4). PGG was also reported to significantly reduce aortic matrix metalloproteinase (MMP) activity in three studies and increase lysyl oxidase (LOX) activity in two studies (Table 4). One study reported no significant effect of PGG on MMP-2 and MMP-9 [13]. Another two studies reported no significant effect of PGG on LOX or the markers of aortic macrophage infiltration [10].

## 4. Discussion

This systematic review of past studies found that the administration of PGG reduced aortic expansion within AAA animal models when measured by direct observation. The findings were not consistent when measured by ultrasound. PGG administered within intravenously injected nanoparticles significantly reduced aortic expansion in studies consistently, whether measured by direct observation or ultrasound. Surprisingly, when PGG treatment was initiated later than when AAA induction commenced (range from 10 to 42 days), it significantly reduced aortic expansion. This was, however, not the case when PGG treatment was started at the time of AAA induction. The findings of other analyses were inconsistent, depending on the method used to measure aortic expansion. A number of important limitations of these prior studies should be noted. Firstly, all studies had a high risk of bias. None of the studies included methods typically thought to be critical in human clinical trials, such as randomization and blinding. Only one study included a sample size calculation [24]. All studies were small and there has been concern that findings from animal models do not translate to AAA patients. This has been particularly reported in relation to doxycycline, but also for fenofibrate, an angiotensin receptor blocker and an angiotensin-converting enzyme inhibitor, which have all been reported to limit aortic expansion in animal models but have not been found to limit AAA growth in clinical trials [7,8,26,27,28].

In addition to the animal experiments reported in this study, there have been other experimental studies reporting the beneficial effects of PGG. In vitro studies have suggested that PGG reduces oxidative stress and MMP secretion and improves the elastic properties of a myoblast cell line [22]. Ex vivo studies of the carotid arteries of mice suggest that PGG protected against elastase-induced artery destruction and limited the mechanical failure of the artery by repairing the elastic lamellae and limiting changes in the mechanical properties of the tissue [29]. A similar ex vivo study using pig aortic samples reported that PGG partially protected against elastase- and collagenase-induced biomechanical changes [30].

One of the key challenges to the use of PGG as a clinical treatment is clarity on the most appropriate route of delivery. None of the animal studies used oral administration, which would be the most straightforward way to administer a medical treatment for AAA. The pharmacokinetics of oral PGG administration are poorly understood, as summarized in detail in a recent review [9]. Low and variable bioavailability of PGG has been reported after oral administration [9]. As illustrated in the included animal studies, a wide range of other routes of administration have been proposed, such as nanoparticles and periadventitial routes, but all are not ideal. Given the low risk (approximately 1% per year) of rupture of small AAAs, any treatment needs to have a good safety profile and, ideally, should be minimally invasive [7].

Despite the limitations of the past animal studies, the positive findings of some studies have encouraged the investigation of PGG as an AAA treatment in patients. In a recent presentation at Aortic Asia, it was announced that PGG delivery via an endovascularly placed balloon to the lumen of the infrarenal aorta is being tested as a treatment of small AAA within a clinical trial. Whether this route of administration, given its relatively invasive nature, is appropriate and feasible to use on a wider scale needs further consideration. Most AAAs contain large volumes of intraluminal thrombus that may interfere with PGG delivery to the aortic wall, and also be at risk of embolization during balloon inflation [31]. Further information on the safety and efficacy of intraluminal PGG is thus required. It is possible that, if this initial clinical trial is encouraging, there could be scope to combine PGG treatment with the endovascular repair of large AAA. A recent systematic review reported a long-term reintervention rate of 18% following endovascular aneurysm repair due to the continued expansion of the AAA sac [32]. The combination of an effective drug and surgical treatment could be a valuable addition to the clinical care of patients with large AAAs. This would need widescale testing to ensure that it is an effective and durable treatment.

A number of limitations of this systematic review should be noted. Firstly, the included studies were small and at high risk of bias. There was insufficient investigation or reporting of aortic rupture to assess this outcome. Finally, and most importantly, since all the current evidence is from animal, ex vivo, or in vitro studies, the clinical relevance of these findings remains unclear. The failure to translate past findings from these types of experiments is again emphasized.

In conclusion, this systematic review suggests inconsistent and low-quality evidence from animal studies that PGG may represent a treatment to restore aortic structure in patients with early-stage AAA. Whether this can translate into a clinically useful treatment is currently unclear, but under investigation by at least one company.

## Figures and Tables

**Table 1:** Characteristics of included studies and animals.

Model	Animals	Age (Months)	Sex	Sample Size †	Sample Size ‡	Modality *	Aortic Diameter Measurement Protocol	Reference
Periadventitial infrarenal aortic elastase	C57BL/6 mice	NR	M	10	10	Ultrasound Photographs of excised aortas (end) and in situ measurements at laparotomy (start)	Systolic maximum inner to inner diameter Maximum outer to outer diameter	[11]
Angiotensin II infusion subcutaneously	LDLR^−/−^ mice	2	M	12	12	Ultrasound	Inner to inner aortic diameter	[23]
Intraluminal infrarenal aortic elastase	Sprague-Dawley rats	NR	M	9	10	Photographs of in situ aortas	Maximum outer to outer diameter	[10]a
Intraluminal infrarenal aortic elastase	Sprague-Dawley rats	NR	M	15 **	15 **	Ultrasound Photographs of in situ aortas	Maximum inner to inner anterior posterior aortic diameter Maximum outer to outer diameter	[10]b
Periadventitial infrarenal aortic calcium chloride	Sprague-Dawley rats	1	M	6	6	Photographs of aortas	Maximum outer to outer diameter	[24]
Periadventitial infrarenal aortic calcium chloride	Sprague-Dawley rats	1	M	6	6	Photographs of in situ aortas	Maximum outer to outer diameter	[12]
Periadventitial infrarenal aortic calcium chloride	Sprague-Dawley rats	NR	F	11	12	Photographs of in situ aortas	Maximum outer to outer diameter	[13]a
Periadventitial infrarenal aortic calcium chloride	Sprague-Dawley rats	NR	F	11	12	Photographs of in situ aortas	Maximum outer to outer diameter	[13]b
Intraluminal infrarenal aortic elastase following balloon dilatation and juxtarenal stenosing cuff	Danish Landrace pigs	NR	F	10	10	Ultrasound	Maximum outer to outer anterior posterior aortic diameter measured in transverse and longitudinal plane	[25]
Periadventitial infrarenal aortic elastase	C57BL/6 mice	2–3.5	NR	8	9	Ultrasound	Inner to inner diameter during systole	[14]a
Periadventitial infrarenal aortic calcium chloride	C57BL/6 mice	2–3.5	NR	8	6	Ultrasound	Inner to inner diameter during systole	[14]b

NR = Not reported; M = Male; F = Female; LDLR^−/−^ = Low-density lipoprotein-receptor-deficient mice maintained on a high fat diet. a/b: Three of the publications included two separate studies that were considered independently; * Represents imaging modality performed at end point; ** One rat was reported to die during the experiment, but outcomes were reported on 15 rats; † Sample size for intervention group; ‡ Sample size for control group.

**Table 2:** Pentagalloyl glucose interventions, controls and outcomes.

Group	Dose	Mode of Delivery	Intervention Commenced †	Duration of Follow-Up ‡	Direct Aortic Percentage	*p*	Direct Aortic Diameter ⸶	*p*	Ultrasound Aortic Percentage	*p*	Ultrasound Aortic Diameter ⸷	*p* Value	AAA Development, *n* (%)	Reference
Intervention	3 mg PGG in 10 mg/kg nanoparticle (on day 14 and 21)	Intravenous	14	14	24.78 ± 15.62 *	<0.0001	NR		9.69 ± 5.24 *	<0.0001	NR		NR	[11]
Control	No administration	NA	NA	14	144.27 ± 28.18 *		NR		110.54 ± 20.37 *		NR		NR	
Intervention	PGG in 10 mg/kg nanoparticles (on day 28 and 42)	Intravenous	28	28	NR		NR		97.75 ± 49.77	<0.05	NR		NR	[23]
Control	Blank in 10 mg/kg nanoparticles (on day 28 and 42)	Intravenous	28	28	NR		NR		182.44 ± 46.55		NR		NR	
Intervention	0.6 mg/mL PGG for 15 min (Once on day 0)	Direct intraluminal delivery	0	28	71.40 ± 46.00	<0.01	3.48 ± 0.91	<0.01	NR		NR		NR	[10]a
Control	2% ethanol, 2.5% DMSO in isotonic saline (Once on day 0)	Direct intraluminal delivery	0	28	159.00 ± 77.50		5.24 ± 1.61		NR		NR		NR	
Intervention	0.6 mg/mL PGG for 15 min (Once on day 0)	Intraluminal delivery via eluting balloon	0	28	183.00 ± 59.10	NS	6.13 ± 1.01	NS	143.00 ± 91.50	NS	5.22 ± 1.06	NS	NR	[10]b
Control	2% ethanol, 2.5% DMSO in isotonic saline (Once on day 0)	Intraluminal delivery via eluting balloon	0	28	149.00 ± 104.00		5.15 ± 1.96		129.20 ± 97.30		4.75 ± 1.91		NR	
Intervention	PGG in 10 mg/kg nanoparticles (on day 42 and 56)	Intravenous	42	42	66.00 ± 21.00	<0.05	NR		NR		NR		6 (100%)	[24]
Control	Blank in 10 mg/kg nanoparticles (on day 42 and 56)	Intravenous	42	42	185.00 ± 25.00		NR		NR		NR		6 (100%)	
Intervention	PGG in 10 mg/kg nanoparticles conjugated with elastin antibody (Once every two weeks from day 10)	Intravenous	10	28	57.00 ± 22.00	<0.05	NR		NR		NR		NR	[12]
Control	Blank in 10 mg/kg nanoparticles (Once every two weeks from day 10)	Intravenous	10	28	158.00 ± 43.00		NR		NR		NR		NR	
Intervention	0.03% *w*/*w* PGG in saline for 15 min (Once on day 0)	Periadventitial application for 15 min	0	28	8.00 ± 7.00	<0.05	1.60 ± 0.09	NR	NR		NR		8 (66.7%) (*p* = NR)	[13]a
Control	Saline (Once on day 0)	Periadventitial application for 15 min	0	28	42.00 ± 10.00		1.90 ± 0.10		NR		NR		2 (18.2%) (*p* = NR)	
Intervention	0.03% *w*/*w* PGG in saline for 15 min (Once on day 28)	Periadventitial application for 15 min	28	28	25.00 ± 7.00	<0.05	NR		NR		NR		NR	[13]b
Control	Saline (Once on day 28)	Periadventitial application for 15 min	28	28	47.10 ± 11.00		NR		NR		NR		NR	
Intervention	25 or 50 mg PGG	Intraluminal delivery	0	28	NR		NR		18.41 ± 2.11	<0.001	12.17 ± 0.13	<0.001	NR	[25]
Control	No administration	Intraluminal delivery	0	28	NR		NR		57.03 ± 10.17		16.26 ± 0.93		NR	
Intervention	0.3% *w*/*w* PGG in saline for 15 min (Once on day 0)	Periadventitial	0	14	NR		NR		137.65 ± 11.98 *	NS	0.85 ± 0.15	NS	NR	[14]a
Control	Saline (Once on day 0)	Periadventitial	0	14	NR		NR		148.98 ± 15.71 *		0.97 ± 0.18		NR	
Intervention	0.3% *w*/*w* PGG in saline for 15 min (Once on day 0)	Periadventitial	0	28	NR		NR		114.48 ± 6.98 *	NS	0.73 ± 0.09	NS	NR	[14]b
Control	Saline (Once on day 0)	Periadventitial	0	28	NR		NR		106.84 ± 3.50 *		0.68 ± 0.07		NR	

NR = Not reported; NA = Not applicable; NS = Not significant; PGG = Pentagalloyl glucose; * Data extracted from graphs or calculated from reported data; † Days after AAA induction was initiated; ‡ Days after intervention commenced; ⸶ Represents increase in aortic diameter using ex vivo measurement; ⸷ Represents increase in aortic diameter using ultrasound measurement; ⸶⸷ Represents primarily ex vivo, and ultrasound measurement if ex vivo was not reported. a/b: Three of the publications included two separate studies that were considered independently.

**Table 3:** Quality assessment of included studies using a modified SYRCLE’s tool for assessing risk of bias.

	Reference	[11]	[23]	[10]a	[10]b	[24]	[12]	[13]a	[13]b	[25]	[14]a	[14]b
Quality Criteria												
Was the allocation sequence adequately generated and applied?		0	0	0	0	0	0	0	0	0	0	0
Were the groups similar at baseline or were they adjusted for confounders in the analysis?		1	1	1	1	1	0	1	1	1	1	1
Was the allocation adequately concealed?		0	0	0	0	0	0	0	0	0	0	0
Were the animals randomly housed during the experiment?		0	0	0	0	0	0	0	0	0	0	0
Were the caregivers and/or investigators blinded from knowledge of which intervention each animal received during the experiment?		0	0	0	0	0	0	0	0	0	0	0
Were animals selected at random for outcome assessment?		0	0	0	0	0	0	0	0	0	0	0
Was the outcome assessor blinded?		0	0	0	1	0	0	0	0	0	0	0
Were incomplete outcome data adequately addressed?		0	0	1	0	1	1	0	0	1	0	0
Are reports of the study free of selective outcome reporting?		0	0	1	0	1	1	0	0	1	0	0
Was the dose of intervention (PGG) justified?		0	0	0	0	0	0	0	0	0	0	0
Was the sample size estimation performed?		0	0	0	0	1	0	0	0	0	0	0
Was the aortic diameter reported within 1 day prior to first allocation to PGG or control and at study completion?		0	1	1	1	0	0	1	1	1	1	1
Was the reproducibility of aortic diameter measurement reported?		0	0	1	1	0	0	0	0	0	0	0
Total Score		1	2	5	4	4	2	2	2	4	2	2
Percentage of possible score		7.69	15.38	38.46	30.77	30.77	15.38	15.38	15.38	30.77	15.38	15.38
**Risk of bias**		**High**	**High**	**High**	**High**	**High**	**High**	**High**	**High**	**High**	**High**	**High**

a/b: Three of the publications included two separate studies that were considered independently.

**Table 4:** Reported effects of PGG on aortic histology and molecular biology findings.

Histology Findings	Molecular Biology Findings	Reference
Suggested aortic elastic fibers were restored in the medial layer (no quantitation); Significantly decreased CD68 positive aortic macrophages (*p* < 0.05)	Suggested decreased MMP-2 (*p* = NR), MMP-9 (*p* = NR) and TGF-b1 (*p* = NR)	[11]
Repaired aortic elastic laminae, improved morphology, and minimal cell infiltration.	Significantly reduced aortic MMP-2 (*p* < 0.05) activity and increased TIMP-1 and -2 (*p* < 0.05). Significantly reduced serum IFN-y and spleen CD68 positive cells (*p* < 0.05)	[23]
Controls had significantly more degraded aortic medial elastic fibers than the PGG-administered group (*p* < 0.01)	mRNA levels of LOX and macrophage marker F4/80 not significantly different between groups	[10]a
NR	mRNA levels of LOX, LOXL1 and macrophage marker F4/80 not significantly different between groups	[10]b
Reduced aortic collagen deposition in PGG-administered compared to controls (not quantitated)	Significant suppression of aortic MMP (*p* < 0.05) and increased LOX (*p* < 0.05) activity compared to controls	[24]
Reduced elastin degradation, calcification, macrophage staining in the adventitial layers (not quantitated)	Significant suppression of aortic MMP (*p* < 0.05) and increased LOX (*p* < 0.05) activity and desmosine content (*p* < 0.05) compared to controls	[12]
Minimal decrease in elastin content and preserved elastic laminar integrity and waviness visually; Significantly greater aortic desmosine (*p* < 0.05)	No significant difference in MMP-2, 9 and TIMP-2. Macrophages and lymphocytes were unaffected (All *p* > 0.05).	[13]a
Improved preservation of elastic laminar integrity and waviness and overall preserved tissue architecture. Aorta media thickness was significantly reduced (*p* < 0.05).	NR	[13]b
Integrity of elastic lamellae was preserved. Light to moderate irregular scattered focal muscle atrophy in the tunica media	NR	[25]
Unchanged levels of calcium and elastin content. Did not exhibit inflammatory characteristic seen in controls.	NR	[14]a
Calcium content was found to be significantly lower in the PGG-treated cohort (*p* = 0.036). No change in elastin content. The extracellular microarchitecture was well preserved (*p* = NR).	NR	[14]b

NR = Not reported; MMP = Matrix metalloproteinase; TIMP = Tissue inhibitor of MMP; CD68 = Cluster of Differentiation 68; LOX = Lysyl oxidase; LOXL1 = Lysyloxidase-like protein 1; IFN-y = Interferon gamma; TGF-b1 = Transforming growth factor beta-1; PGG = Pentagalloyl glucose. a/b: Three of the publications included two separate studies that were considered independently.

## Data Availability

Not applicable.

## Supplementary Materials

The following are available online at https://www.mdpi.com/article/10.3390/biomedicines9101442/s1, Figure S1: Meta-analysis of studies testing the effect of pentagalloyl glucose on AAA incidence. RR = Relative risk; Ne = Number of animals in experimental group; Nc = Number of animals in control group; CI = Confidence interval, Table S1: Leave-one-out sensitivity analysis of studies reporting aortic expansion through direct measurement.
