# Supplementary material for: A Systematic Review and Meta-Analysis of the Effect of Pentagalloyl Glucose Administration on Aortic Expansion in Animal Models
Source: Biomedicines. 2021 Oct 11;9(10):1442. doi: 10.3390/biomedicines9101442 (PMC8533208; doi:10.3390/biomedicines9101442)
Supplement: Supplementary file 1 [file biomedicines-09-01442-s001.zip › biomedicines-1413991-supplementary.pdf]

Supplementary material

Supplementary table S1: Leave-one-out sensitivity analysis of studies reporting aortic expansion through direct measurement

Sensitivity analysis	Effect	LLCI	ULCI	I2	Heterogeneity contribution	Influence effect size
Omitting Dhital 2020	-56.608	-101.76	-11.456	0.894	17.591	7.354
Omitting Isenburg 2007b	-74.161	-120.985	-27.336	0.901	17.579	3.861
Omitting Isenburg 2007a	-71.912	-120.305	-23.519	0.912	12.86	0.426
Omitting Schack 2020b	-79.939	-114.861	-45.018	0.915	12.272	3.173
Omitting Nosoudi 2016 (1)	-56.968	-102.245	-11.691	0.916	10.044	2.09
Omitting Nosoudi 2016 (2)	-60.389	-108.294	-12.485	0.929	1.512	0.129
Omitting Schack 2020a	-62.932	-111.674	-14.19	0.93	0.141	0.005

LLCI = Lower limit confidence intervals; ULCI = Upper limit confidence intervals

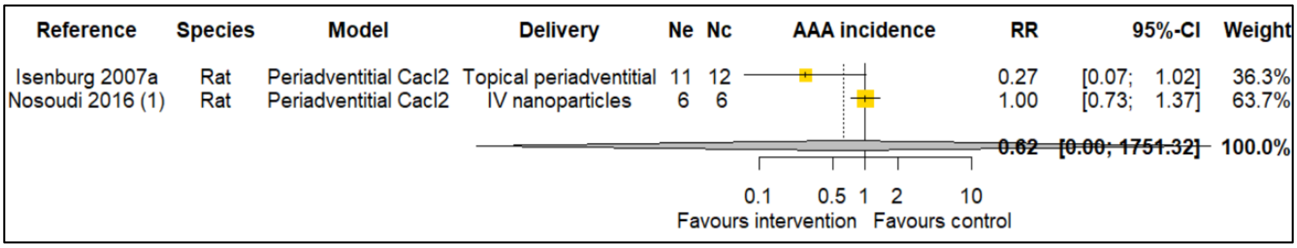

Supplementary figure S1: Meta-analysis of studies testing the effect of pentagalloyl glucose on AAA incidence. RR = Relative risk; Ne = Number of animals in experimental group; Nc = Number of animals in control group; CI = Confidence interval.
